# Diabetes Mellitus and Cognitive Decline: A Systematic Review Exploring the Link to Dementia and Neurodegenerative Diseases

**DOI:** 10.7759/cureus.80415

**Published:** 2025-03-11

**Authors:** Abdul Saboor Khaliq, Aroon Kumar, Mahnoor Khan, Zarin Nudar Rodoshi

**Affiliations:** 1 Internal Medicine, Allama Iqbal Medical College, Lahore, PAK; 2 Medicine and Surgery, Khairpur Medical College, Khairpur, PAK; 3 Medicine and Surgery, Fazaia Medical College, Islamabad, PAK; 4 Medical Education, Mymensingh Medical College, Dhaka, BGD

**Keywords:** alzheimer’s disease, cognitive decline, dementia, diabetes mellitus, glucose-lowering therapies, meta-analysis, systematic review, tau biomarkers

## Abstract

This systematic review explores the association between diabetes mellitus (DM) and cognitive decline, with a particular focus on neurodegenerative diseases such as dementia and Alzheimer’s disease (AD). A comprehensive search strategy, conducted in accordance with Preferred Reporting Items for Systematic reviews and Meta-Analyses (PRISMA) guidelines, identified and selected nine meta-analyses of high relevance and methodological rigor. These studies encompassed diverse populations, including stroke survivors, apolipoprotein E (APOE) ɛ4 carriers, and individuals with metabolic syndrome, and examined both interventions, such as glucose-lowering therapies, and risk factors, including hypoglycemia and poor glycemic control. Key findings indicate that diabetes is a significant risk factor for cognitive decline, with strong associations observed between impaired glucose metabolism and elevated tau biomarkers. Glucose-lowering therapies, particularly sodium-glucose cotransporter-2 (SGLT-2) inhibitors and metformin, demonstrated potential neuroprotective effects, reducing the risk of dementia and cognitive impairment. However, heterogeneity among the studies and variability in study designs highlight the need for further high-quality research to validate these findings and elucidate underlying mechanisms. This review underscores the importance of integrating cognitive health into diabetes management and highlights the potential of targeted interventions to mitigate the cognitive burden associated with diabetes.

## Introduction and background

Diabetes mellitus (DM) has emerged as a global health challenge, affecting more than 500 million individuals worldwide, with its prevalence expected to rise significantly in the coming decades [1]. While the metabolic and cardiovascular complications of diabetes are well-established, its impact on cognitive function and the progression to neurodegenerative diseases is gaining increasing recognition. Both type 1 and type 2 diabetes are linked to an elevated risk of cognitive decline, mild cognitive impairment (MCI), and dementia, including Alzheimer’s disease (AD) and vascular dementia (VD) [2]. The intricate interplay between chronic hyperglycemia, insulin resistance, inflammation, oxidative stress, and advanced glycation end products (AGEs) creates a neurotoxic environment, ultimately contributing to neuronal damage and cognitive deficits [3].

Emerging evidence suggests that diabetes-related complications, such as hypoglycemia, diabetic retinopathy, and cerebrovascular events, exacerbate the risk of cognitive impairments. Moreover, genetic predispositions, such as APOE ε4 carrier status, have been shown to amplify the susceptibility of diabetic patients to dementia [4]. Additionally, the role of diabetes management, including the use of glucose-lowering medications like sodium-glucose cotransporter-2 (SGLT2) inhibitors and metformin, in modifying cognitive outcomes is a critical area of investigation. These medications not only improve glycemic control but may also exert neuroprotective effects by reducing inflammation, improving cerebral glucose metabolism, and enhancing vascular integrity.

Given the increasing burden of diabetes and its complex association with cognitive decline, a comprehensive understanding of the underlying mechanisms and potential therapeutic interventions is vital [5]. Systematic reviews and meta-analyses offer valuable insights by synthesizing data from diverse studies, enabling a nuanced exploration of diabetes-related cognitive decline and its interplay with neurodegenerative diseases. This systematic review aims to evaluate existing meta-analyses to elucidate the relationship between diabetes and cognitive decline, identify modifiable risk factors, and assess the impact of various therapeutic strategies on neurocognitive outcomes.

The population of interest for this systematic review includes individuals diagnosed with diabetes mellitus, encompassing both type 1 and type 2 diabetes, regardless of whether they exhibit pre-existing cognitive impairments or neurodegenerative conditions. The interventions focus on the management of diabetes through glucose-lowering therapies, particularly highlighting the use of metformin, SGLT2 inhibitors, and other treatment strategies aimed at achieving optimal glycemic control and potentially mitigating cognitive decline. The comparisons involve diabetic individuals who are either not receiving specific glucose-lowering therapies, receiving alternative treatments, or belonging to non-diabetic populations for a broader perspective. The outcomes of interest center on cognitive parameters, including the risk and progression of MCI and dementia, such as Alzheimer’s disease or vascular dementia. Additionally, the review will consider neurodegenerative biomarkers, such as amyloid and tau protein levels, as well as cognitive performance metrics to comprehensively evaluate the interlink between diabetes, its management, and cognitive decline. This framework provides a structured approach to investigate the potential pathways and therapeutic impacts associated with diabetes-related cognitive impairments.

## Review

Materials and methods

Search Strategy

The search strategy for this systematic review was specifically designed to identify and include only meta-analyses that explored the association between DM, cognitive decline, and neurodegenerative diseases, such as dementia and AD. Following Preferred Reporting Items for Systematic reviews and Meta-Analyses (PRISMA) guidelines [6], a comprehensive search was conducted across multiple databases, including PubMed, Embase, Cochrane Library, and Web of Science, using a combination of Medical Subject Headings (MeSH) and keywords such as “diabetes mellitus,” “cognitive impairment,” “dementia,” “Alzheimer's disease,” “tau,” “amyloid-β,” and “meta-analysis.” Filters were applied to include only meta-analyses published in English up to the most recent date. Studies were screened for relevance based on predefined inclusion and exclusion criteria, aligned with the Population, Intervention, Comparison, and Outcome (PICO) framework, focusing on population, intervention, comparison, and outcomes. Additional meta-analyses were identified through manual screening of reference lists of included articles. This targeted approach ensured the selection of high-quality evidence that synthesized findings from multiple studies, providing a robust basis for analyzing the interplay between diabetes and cognitive health. All included meta-analyses underwent rigorous quality assessment to ensure methodological reliability.

Eligibility Criteria

The eligibility criteria for this systematic review were established to ensure the inclusion of high-quality meta-analyses that comprehensively explored the association between DM, cognitive decline, and neurodegenerative diseases such as dementia and AD. Only meta-analyses published in English and available in full text were included, with publication dates extending up to the most recent updates in 2024. Studies were required to focus on populations with DM (type 1 or type 2) and report on cognitive outcomes such as MCI, dementia, AD, or relevant biomarkers (e.g., tau and amyloid-β). Meta-analyses that evaluated the effects of interventions, such as glucose-lowering therapies (e.g., SGLT-2 inhibitors, metformin), or examined risk factors like hypoglycemia, glycemic control, or genetic predispositions (e.g., APOE ε4 carriers), were prioritized.

Exclusion criteria included meta-analyses that did not focus on diabetes or cognitive outcomes, those that combined data from non-diabetic populations without stratification, narrative reviews, primary studies, or non-meta-analytical systematic reviews. Studies with unclear methodology or insufficient statistical data for pooled analysis were also excluded. This rigorous eligibility framework ensured that the included meta-analyses provided robust and reliable evidence, forming the foundation for exploring the complex interplay between diabetes, cognitive decline, and neurodegeneration.

Data Extraction

Data extraction for this systematic review was performed using a standardized template to ensure consistency and accuracy across all included meta-analyses. Key details extracted from each study included authorship, publication year, population characteristics (e.g., diabetes type, comorbidities, and sample size), intervention details (e.g., glucose-lowering therapies or risk factors like glycemic control and genetic predispositions), comparison groups, cognitive outcomes (e.g., MCI, dementia, AD, or biomarkers like tau and amyloid-β), study type, and key findings such as RRs, HRs, or ORs. Additional data on study methodology, such as search strategies, statistical approaches, and heterogeneity measures, were also collected. Two independent reviewers conducted the data extraction process to minimize bias, with discrepancies resolved through discussion or a third reviewer. This rigorous approach ensured that the extracted data provided a comprehensive and reliable basis for synthesizing the evidence on the relationship between diabetes and cognitive decline.

Data Analysis and Synthesis

Data analysis and synthesis were conducted to comprehensively evaluate the findings from the included meta-analyses and to identify overarching trends and patterns. Key outcomes, such as RRs, HRs, ORs, and standardized mean differences, were extracted and summarized to assess the relationship between DM and cognitive outcomes, including MCI, dementia, AD, and associated biomarkers like tau and amyloid-β. Results were synthesized to explore how specific factors, such as glycemic control, glucose-lowering therapies, and genetic predispositions, modulate the risk of cognitive decline. Subgroup analyses were conducted where applicable, focusing on differences in population characteristics (e.g., APOE ε4 carriers, stroke survivors) or interventions (e.g., SGLT-2 inhibitors, metformin). Heterogeneity across the included meta-analyses was considered, and potential sources of variation were identified. Narrative synthesis was also employed to contextualize findings and provide a cohesive interpretation of the data, ensuring that the analysis highlighted both consistencies and gaps in the current evidence. This rigorous approach enabled a nuanced understanding of the complex interplay between diabetes and cognitive health.

Results

Study Selection Process

**Figure 1:**
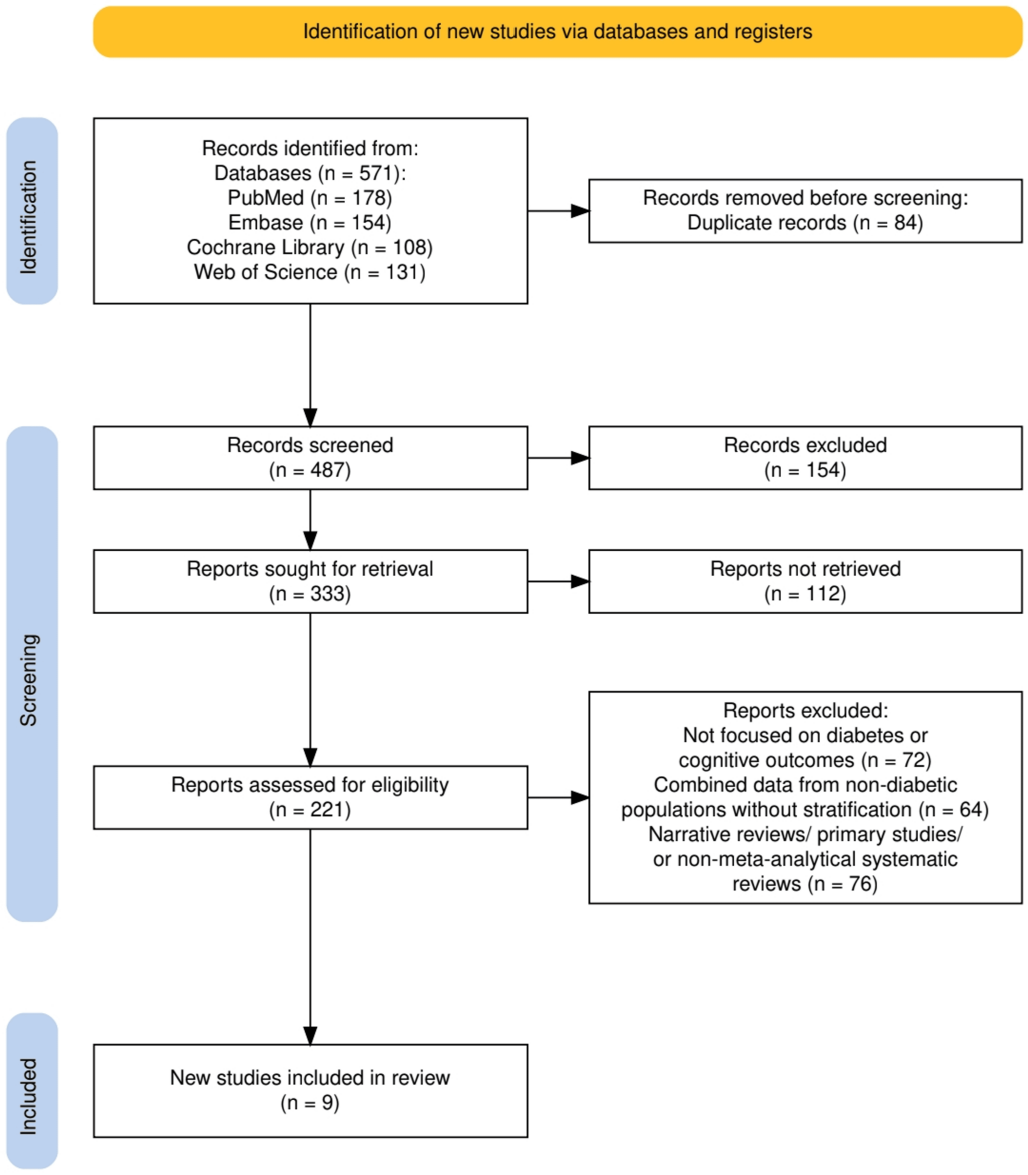
The PRISMA flowchart represents the study selection process. PRISMA: Preferred Reporting Items for Systematic reviews and Meta-Analyses.

The study selection process, illustrated in Figure 1, involved a systematic and meticulous approach adhering to PRISMA guidelines. A total of 571 records were initially identified from four major databases: PubMed (178), Embase (154), Cochrane Library (108), and Web of Science (131). After removing 84 duplicate records, 487 unique records were screened based on titles and abstracts. From these, 154 records were excluded for not meeting the inclusion criteria. The remaining 333 reports were sought for retrieval, but 112 could not be retrieved due to access limitations or availability issues. Of the 221 reports assessed for eligibility, 212 were excluded for reasons including a lack of focus on diabetes or cognitive outcomes (72), the inclusion of non-diabetic populations without stratification (64), or being narrative reviews, primary studies, or non-meta-analytical systematic reviews (76). Ultimately, nine studies meeting all inclusion criteria were selected for the systematic review, ensuring a high-quality and focused synthesis of evidence.

Characteristics of the Selected Studies

The selected studies, as presented in Table 1, provide a comprehensive examination of the relationship between DM, cognitive decline, and neurodegenerative diseases, using meta-analytical approaches to synthesize data from diverse populations. The studies include individuals with DM, MCI, dementia, and genetic predispositions, such as APOE ε4 carriers. Interventions explored range from glucose-lowering therapies, such as SGLT-2 inhibitors, DPP-4 inhibitors, and metformin, to analyses of risk factors like glycemic control, hypoglycemia, and metabolic syndrome. Key outcomes assessed include the risk of cognitive impairment, dementia (including AD and VD), and associated biomarkers like tau and amyloid-β. The systematic review and meta-analysis methodologies employed across the studies ensure robust statistical synthesis, while subgroup analyses and stratifications provide additional insights into modifiable and non-modifiable risk factors. These characteristics reflect a well-rounded selection of studies that contribute significantly to understanding the complex interplay between diabetes and cognitive health.

**Table 1:** Characteristics of the included studies. TIA: Transient Ischemic Attack; PSCI: Post-Stroke Cognitive Impairment; PSD: Post-Stroke Dementia; T2D: Type 2 Diabetes; GLDs: Glucose-Lowering Drugs; DPP-4: Dipeptidyl Peptidase-4; GLP-1RAs: Glucagon-Like Peptide-1 Receptor Agonists; SGLT2: Sodium-Glucose Cotransporter-2; AD: Alzheimer’s Disease; VD: Vascular Dementia; CF: Cognitive Frailty; HbA1c: Glycated Hemoglobin; APOE ɛ4: Apolipoprotein E epsilon 4; RCTs: Randomized Controlled Trials; SMD: Standardized Mean Difference; MCI: Mild Cognitive Impairment; MetS: Metabolic Syndrome; DM: Diabetes Mellitus.

Authors & Year	Population	Intervention	Comparison	Outcome(s)	Study Type	Key Findings
Filler J et al. (2024) [7]	Stroke survivors (ischemic, hemorrhagic, and TIA) with baseline cognitive and clinical data, followed for at least 3 months.	Identification of risk factors for post-stroke cognitive impairment (PSCI) and dementia (PSD), focusing on modifiable factors such as diabetes, atrial fibrillation, and cerebral small vessel disease markers.	Comparison of stroke survivors with and without PSCI/PSD and stratification by the presence of risk factors.	Risk factors for PSCI and PSD, including baseline cognitive impairment, diabetes (RR: 1.29 for PSCI; 1.38 for PSD), atrial fibrillation (RR: 1.29 for PSCI), white matter hyperintensities (RR: 1.51 for PSCI; 1.55 for PSD), and others (e.g., lower education, brain atrophy).	Systematic review and meta-analysis (113 articles, 89 studies, 160,783 patients).	Diabetes, atrial fibrillation, and markers of cerebral small vessel disease are significant and treatable risk factors for PSCI and PSD. Baseline cognitive impairment is the strongest predictor of cognitive decline after stroke. The study emphasizes the importance of targeted interventions for these factors to reduce the burden of cognitive impairment in stroke survivors.
Tang H et al. (2023) [8]	Adults with type 2 diabetes (T2D), involving 819,511 individuals.	Use of newer glucose-lowering drugs (GLDs) including DPP-4 inhibitors, GLP-1 receptor agonists (GLP-1RAs), and SGLT2 inhibitors.	Non-users of these drugs or users of other GLDs.	Risk of all-cause dementia, Alzheimer’s disease (AD), and vascular dementia (VD).	Systematic review and meta-analysis (10 studies from 9 articles).	Newer GLDs were associated with reduced risk of all-cause dementia in T2D patients: SGLT2 inhibitors (RR: 0.62; 95% CI, 0.39-0.97), GLP-1RAs (RR: 0.72; 95% CI, 0.54-0.97), and DPP-4 inhibitors (RR: 0.84; 95% CI, 0.74-0.94). Significant reduction in VD risk was observed with DPP-4 inhibitors (RR: 0.59; 95% CI, 0.47-0.75), but not AD. Findings should be interpreted cautiously due to observational nature and heterogeneity.
Peng J et al. (2023) [9]	Diabetic patients in China, assessed for cognitive frailty (CF).	Analysis of prevalence and related factors for cognitive frailty, with risk and protective factors identified (e.g., depression, malnutrition, advanced age, regular exercise).	Comparison of diabetic patients with and without CF, including subgroups by hospital vs. community settings and gender.	Prevalence of CF in diabetic patients (25.8%, 95% CI: 19.7–31.9%), and associated factors (e.g., depression, malnutrition, advanced age, high HbA1c as risk factors; regular exercise and high education as protective factors).	Systematic review and meta-analysis (18 studies included).	Cognitive frailty was prevalent in 25.8% of Chinese diabetic patients. Risk factors included depression, malnutrition, advanced age, chronic disease burden, and high HbA1c levels, while regular exercise and higher education were protective. Hospital prevalence was higher than in the community, and CF was more common in women. Early screening and intervention are recommended.
Youn YJ et al. (2024) [10]	Patients with diabetes mellitus, including those with mild cognitive impairment or dementia.	Use of sodium-glucose cotransporter-2 (SGLT-2) inhibitors.	Non-users of SGLT-2 inhibitors.	Risk of dementia (Hazard ratio: 0.68, 95% CI: 0.50–0.92) and cognitive function improvement (Standardized Mean Difference: 0.88, 95% CI: 0.32–1.44).	Systematic review and meta-analysis (studies published up to August 2023).	SGLT-2 inhibitors were associated with a significantly lower risk of dementia and improved cognitive function scores in diabetic patients, particularly in those with mild cognitive impairment or dementia. The study highlights the potential neuroprotective role of SGLT-2 inhibitors and emphasizes the need for well-controlled clinical trials to validate these findings.
Rashtchian A et al. (2024) [11]	APOE ε4 carriers with type 2 diabetes (T2D); total population: 42,390 from nine cohorts and seven cross-sectional studies.	Analysis of the impact of diabetes mellitus on dementia risk in APOE ε4 carriers.	Non-diabetic APOE ε4 carriers.	Risk of dementia in diabetic APOE ε4 carriers (Hazard Ratio: 1.48, 95% CI: 1.36–1.60); dementia frequency of 30% (95% CI: 0.15–0.48).	Meta-analysis (articles published up to September 2023).	Diabetes increases the risk of dementia by 48% in APOE ε4 carriers. The study observed no significant heterogeneity or publication bias (Egger's test, p = 0.2). The findings highlight the interplay between genetic predisposition (APOE ε4) and diabetes in elevating dementia risk, emphasizing the need for further large-scale studies.
Campbell JM et al. (2018) [12]	Diabetic patients, including those at risk of or diagnosed with dementia, Alzheimer’s disease, or cognitive impairment; total of 14 studies (7 cohort, 4 cross-sectional, 2 RCTs, 1 case-control).	Use of metformin as a first-line antihyperglycemic medication.	Non-metformin users (diabetic and non-diabetic controls).	Reduced prevalence of cognitive impairment (OR: 0.55, 95% CI: 0.38–0.78); reduced dementia incidence (HR: 0.76, 95% CI: 0.39–0.88); non-significant effect on Mini-Mental State Examination scores.	Systematic review and meta-analysis.	Metformin use was associated with significantly lower risk of cognitive impairment and dementia in diabetic patients. Both RCTs in the review demonstrated neuroprotective effects. Negative effects were noted in some studies, possibly linked to metformin-induced vitamin B12 deficiency. Metformin remains a recommended first-line therapy for diabetes patients at risk of cognitive decline. Its use in non-diabetics for dementia prevention is not supported by evidence.
Mu Z et al. (2024) [13]	Older diabetic patients (≥45 years), including 6,045 individuals across 7 studies.	Analysis of cognitive performance in patients with hypoglycemic episodes.	Diabetic patients without hypoglycemic episodes.	Cognitive performance: general intelligence (SMD: 0.58, 95% CI: 0.88–0.28), memory performance (SMD: 0.19, 95% CI: 0.29–0.09), and psychomotor function (SMD: 0.51, 95% CI: 0.38–0.63).	Meta-analysis (7 studies).	Older diabetic patients with hypoglycemic episodes demonstrated significantly worse cognitive performance, including general intelligence, memory, and psychomotor function, compared to those without hypoglycemia. Findings highlight the potential impact of hypoglycemia on cognitive decline in elderly diabetic populations.
Pal K et al. (2018) [14]	People with mild cognitive impairment (MCI), diabetes, prediabetes, or metabolic syndrome (MetS); 6,865 participants from 12 studies.	Analysis of the progression of MCI to dementia in individuals with diabetes, prediabetes, or MetS.	Comparison with individuals without diabetes, prediabetes, or MetS.	Risk of progression from MCI to dementia: diabetes (OR: 1.53, 95% CI: 1.20–1.97), MetS (OR: 2.95, 95% CI: 1.23–7.05), and combined diabetes/MetS (OR: 1.67, 95% CI: 1.27–2.19).	Systematic review and meta-analysis (12 studies).	Diabetes and MetS were associated with significantly higher risk of progression from MCI to dementia. Longer diabetes duration and retinopathy increased risk, while statins and oral hypoglycemic agents reduced risk. Cardiovascular risk factors and lifestyle changes were critical in mitigating dementia risk in this population.
Van Gils V et al. (2024) [15]	Individuals with and without diabetes mellitus (DM); total of 11,694 participants from 37 studies.	Analysis of glucose metabolism measures (e.g., glycated hemoglobin, fasting blood glucose, insulin resistance indices) and DM status in relation to Alzheimer’s disease (AD) biomarkers (amyloid-β and tau).	Individuals with normal glucose metabolism and no DM status.	DM and impaired glucose metabolism were associated with higher tau biomarkers (r = 0.11, 95% CI: 0.03–0.18, p = 0.008) but not amyloid-β biomarkers (r = -0.06, 95% CI: -0.13–0.01, p = 0.08).	Systematic review and meta-analysis (37 studies).	DM and impaired glucose metabolism are significantly associated with higher tau biomarkers, particularly in population settings. Amyloid-β biomarkers were not associated with DM overall but showed lower levels in memory clinic settings, studies with higher dementia prevalence, or lower cognitive scores. Findings highlight the link between glucose metabolism, tau pathology, and AD diagnostics.

Quality Assessment

The quality assessment of the selected studies, as summarized in Table 2, highlights a generally robust methodological framework, with many studies adhering to AMSTAR 2 criteria [16] and demonstrating high overall quality. Studies with registered protocols, such as those utilizing PROSPERO [17], comprehensive search strategies, and rigorous meta-analytical methods, were rated as high quality due to their adherence to PRISMA guidelines and effective management of heterogeneity. These studies often included thorough subgroup analyses, appropriate risk of bias assessments, and transparent reporting of findings. However, several studies were rated as moderate quality due to limitations such as the inclusion of observational designs, the absence of protocol registration, or moderate heterogeneity. Despite these limitations, most studies provided valuable insights through detailed data synthesis and interpretation. The quality assessment underscores the reliability of the findings while identifying areas for methodological improvement, such as ensuring protocol registration and minimizing heterogeneity in future research.

**Table 2:** The quality assessment of the included studies. PRISMA: Preferred Reporting Items for Systematic reviews and Meta-Analyses.

Authors & Year	AMSTAR 2 Criteria	Overall Quality	Comments
Filler J et al. (2024) [7]	Registered protocol (PROSPERO), comprehensive search strategy, inclusion of risk of bias assessment, appropriate meta-analysis, low heterogeneity.	High	Study follows PRISMA guidelines with strong methodology, registered protocol, and robust analysis. Low heterogeneity supports reliability.
Tang H et al. (2023) [8]	Comprehensive search strategy, appropriate inclusion/exclusion criteria, clear risk of bias assessment, moderate heterogeneity, observational studies included.	Moderate	Observational studies reduce reliability, though the analysis is rigorous and includes a clear search strategy.
Peng J et al. (2023) [9]	Detailed database search, subgroup analysis, clear assessment of risk factors, but no clear registration of a protocol. Moderate heterogeneity.	Moderate	High-quality synthesis but lacks protocol registration, which reduces reproducibility.
Youn YJ et al. (2024) [10]	Registered protocol, robust meta-analysis, low heterogeneity, comprehensive inclusion of studies, thorough subgroup analysis.	High	Clear methodology with well-controlled heterogeneity and thorough data interpretation. Registered protocol strengthens reliability.
Rashtchian A et al. (2024) [11]	Registered protocol, moderate heterogeneity, inclusion of risk of bias assessment, rigorous meta-analysis, clear reporting of Egger's test.	High	Comprehensive study with appropriate controls for bias and heterogeneity. Robust findings supported by statistical methods.
Campbell JM et al. (2018) [12]	Registered protocol, comprehensive search, mix of study designs (cohort, cross-sectional, RCTs), potential heterogeneity, some negative/neutral results discussed narratively.	Moderate	Inclusion of diverse study designs limits reliability, though the protocol registration and robust synthesis add value.
Mu Z et al. (2024) [13]	Clear search strategy, appropriate meta-analysis methods, moderate heterogeneity (I² < 60%), but lacks explicit protocol registration.	Moderate	High-quality data synthesis but reduced reliability due to missing protocol registration.
Pal K et al. (2018) [14]	Comprehensive search strategy, clear inclusion/exclusion criteria, moderate heterogeneity, lack of protocol registration.	Moderate	High-quality analysis, but protocol registration would improve reliability.
Van Gils V et al. (2024) [15]	Registered protocol, robust meta-regression, appropriate methods for assessing heterogeneity, thorough evaluation of tau and amyloid-β biomarkers.	High	Strong methodology with detailed statistical analysis. Protocol registration ensures reproducibility.

Discussion

This systematic review synthesized findings from multiple meta-analyses to explore the relationship between DM and cognitive decline, including dementia and AD. The evidence highlights a consistent link between diabetes and an increased risk of cognitive impairments and neurodegenerative diseases. Studies by Filler J et al. [7] and Rashtchian A et al. [11] demonstrated that diabetes is a significant risk factor for cognitive impairment and dementia, especially among stroke survivors (RR: 1.29, 95% CI: 1.12-1.48) and APOE ε4 carriers (RR: 1.48, 95% CI: 1.36-1.60), respectively. Additionally, markers of small vessel disease, such as white matter hyperintensities (RR: 1.51 for post-stroke cognitive impairment, 95% CI: 1.32-1.72; RR: 1.55 for post-stroke dementia, 95% CI: 1.41-1.71), were identified as modifiable contributors to post-stroke cognitive decline. Preventive strategies for reducing white matter hyperintensities include optimizing glycemic control, managing hypertension, reducing vascular risk factors, and promoting lifestyle modifications such as regular physical activity and dietary interventions.

Therapies such as SGLT-2 inhibitors and metformin were associated with promising neuroprotective effects. Youn YJ et al. [10] found that SGLT-2 inhibitors reduced dementia risk (HR: 0.68, 95% CI: 0.50-0.92) and improved cognitive function scores in diabetic patients, while Campbell JM et al. [12] reported that metformin significantly lowered the odds of cognitive impairment (OR: 0.55, 95% CI: 0.38-0.78) and dementia (HR: 0.76, 95% CI: 0.39-0.88). Furthermore, Van Gils V et al. [15] demonstrated a clear association between impaired glucose metabolism and increased tau biomarkers, underscoring the mechanistic link between diabetes and AD pathology. However, while these associations are robust, causality cannot be definitively established due to potential confounding factors, study design heterogeneity, and variability in patient populations. Most included studies are observational or meta-analyses of non-randomized trials, limiting causal inferences. Further randomized controlled trials (RCTs) are necessary to confirm whether these interventions directly influence cognitive decline or if their effects are mediated through improved metabolic control and vascular health. Collectively, these findings address the research hypothesis by illustrating the multifactorial interplay between diabetes, its treatments, and cognitive outcomes, emphasizing the importance of targeted interventions in at-risk populations.

The findings of this systematic review align with prior studies that have established a strong association between DM and cognitive decline, including dementia and AD [18]. Consistent with earlier meta-analyses, such as those by Chatterjee S et al. [19] and Xue M et al. [20], which demonstrated an increased risk of cognitive impairment in diabetic patients, this review further highlights the contributory role of diabetes-related factors, such as poor glycemic control and vascular complications, in accelerating neurodegenerative processes. Notably, our review corroborates the protective effects of glucose-lowering medications, as observed in prior studies, with SGLT-2 inhibitors and metformin showing significant reductions in the risk of dementia [21]. These findings reinforce the emerging evidence suggesting that targeted pharmacological interventions can mitigate cognitive decline in diabetic populations by addressing underlying metabolic and vascular dysfunction [22].

However, discrepancies with existing literature were also observed. While previous studies have largely emphasized the role of amyloid-β in AD pathophysiology, our findings, particularly from Van Gils V et al. [15], indicate that diabetes and impaired glucose metabolism are more closely linked with tau pathology rather than amyloid-β accumulation. This divergence may reflect differences in population characteristics, biomarker assessment techniques, or study designs across included research. Additionally, while some studies have reported inconsistent effects of metformin on cognitive outcomes, potentially due to confounding by vitamin B12 deficiency, our review supports its neuroprotective potential in diabetic patients, as also suggested by Campbell JM et al. [12]. By synthesizing these findings, this review contributes to closing critical knowledge gaps, emphasizing the need for personalized therapeutic approaches targeting modifiable risk factors and specific pathophysiological pathways in diabetes-related cognitive decline.

The findings of this review underscore the multifaceted relationship between DM and cognitive decline, revealing both direct and indirect mechanisms that contribute to neurodegeneration [23]. Diabetes appears to exacerbate cognitive impairment through pathways such as chronic hyperglycemia, insulin resistance, and inflammation, which together promote vascular damage, oxidative stress, and the accumulation of tau proteins, as evidenced by Van Gils V et al. [15]. These biological factors not only disrupt neuronal integrity but also amplify the impact of comorbidities, such as atrial fibrillation and white matter hyperintensities, highlighted by Filler J et al. [7], on cognitive outcomes. Furthermore, the neuroprotective effects of newer glucose-lowering drugs, particularly SGLT-2 inhibitors and metformin, suggest that addressing metabolic dysfunction can reduce the risk of dementia, possibly by improving insulin signaling and reducing systemic inflammation, as observed in studies by Youn YJ et al. [10] and Campbell JM et al. [12]. These results are highly relevant for clinical practice, emphasizing the need for comprehensive diabetes management strategies that not only target glycemic control but also mitigate cognitive risks through early identification and tailored interventions [24].

The findings of this review have significant clinical, public health, and research implications, emphasizing the urgent need to integrate cognitive health assessments into routine diabetes care. Clinically, the demonstrated association between diabetes and cognitive decline highlights the importance of early screening for cognitive impairments, particularly in high-risk populations such as stroke survivors, APOE ε4 carriers, and those with poor glycemic control. Public health strategies should prioritize widespread education on the risks of diabetes-related neurodegeneration and promote interventions such as intensive cardiovascular risk management, lifestyle modifications, and the use of neuroprotective glucose-lowering therapies like SGLT-2 inhibitors and metformin. Research efforts should focus on conducting large-scale randomized controlled trials to validate the neuroprotective effects of these therapies and explore the underlying mechanisms linking diabetes to tau pathology and other biomarkers. These findings also underscore the need for developing targeted interventions tailored to individual risk profiles, potentially transforming diabetes management into a holistic approach that incorporates cognitive preservation as a key therapeutic goal.

This systematic review has several strengths, including a comprehensive search strategy that spanned multiple databases and adhered to PRISMA guidelines, ensuring the inclusion of high-quality studies. The use of robust statistical methods, including meta-analyses and subgroup analyses, allowed for meaningful synthesis of data from diverse study populations. However, some limitations must be acknowledged. Heterogeneity was observed across the included studies, stemming from variability in study designs (e.g., observational studies versus RCTs) and differences in populations, interventions, and outcome measures. Additionally, while many studies had registered protocols, a lack of consistent protocol registration among some included studies may introduce bias. Geographic and demographic biases, such as the overrepresentation of Chinese populations in certain analyses, may limit the generalizability of findings. These limitations suggest that while the results provide valuable insights into the relationship between diabetes and cognitive decline, they should be interpreted with caution, and further research is needed to confirm the findings in more diverse and standardized settings.

This review highlights several gaps in knowledge that warrant further investigation. While the findings suggest strong associations between diabetes and cognitive decline, there is a need for high-quality RCTs to validate the neuroprotective effects of glucose-lowering therapies, such as SGLT-2 inhibitors and metformin, in diverse populations. The role of specific subpopulations, such as APOE ε4 carriers and individuals with coexisting metabolic syndrome or cardiovascular disease, remains underexplored and could provide critical insights into tailored interventions [25]. Additionally, the observed link between diabetes and tau pathology, rather than amyloid-β, raises questions about the mechanistic pathways underlying this association, which could be addressed through biomarker-focused longitudinal studies. Future research should also investigate the impact of early lifestyle modifications, such as exercise and dietary interventions, on reducing cognitive decline among diabetic patients, as well as the role of sex- and age-specific differences in disease progression. These efforts will help establish causal relationships and refine prevention and management strategies for diabetes-related neurodegeneration.

## Conclusions

This systematic review underscores the significant relationship between diabetes mellitus and cognitive decline, including dementia and Alzheimer’s disease, emphasizing the critical role of metabolic and vascular factors in neurodegeneration. The findings highlight the potential of glucose-lowering therapies, such as SGLT-2 inhibitors and metformin, to reduce cognitive risks, alongside the importance of early interventions targeting modifiable factors like glycemic control, cardiovascular risk, and lifestyle changes. While the results provide valuable insights, they also reveal gaps in knowledge that call for further high-quality research to validate these associations and explore targeted approaches for at-risk populations. These findings not only enhance our understanding of the complex interplay between diabetes and cognitive health but also offer actionable pathways for improving clinical care, public health strategies, and research priorities to mitigate the growing burden of diabetes-related neurodegeneration.
